# Effect of Fe and C Contents on the Microstructure and High-Temperature Mechanical Properties of IN625 Alloy Processed by Laser Powder Bed Fusion

**DOI:** 10.3390/ma15196606

**Published:** 2022-09-23

**Authors:** Alena Kreitcberg, Vladimir Brailovski

**Affiliations:** Department of Mechanical Engineering, École de technologie supérieure, Montreal, QC H3C 1K3, Canada

**Keywords:** additive manufacturing, laser powder bed fusion, microstructure, mechanical properties, ductility loss, IN625

## Abstract

Two alloys with different Fe and C contents were studied to assess the influence of their compositions on the microstructure and mechanical properties of Ni-based Inconel 625 superalloy processed by laser powder bed fusion and subjected to stress relief annealing (870 °C) and a solution treatment (1120 °C). It was concluded that the alloy with a higher Fe content (~4 wt.% as compared to ~1 wt.%) manifests a greater propensity to segregate Nb and Mo elements during printing and form δ phase particles during the stress relief annealing. On the other hand, the alloy with a higher C content (~0.04 wt.% compared to ~0.02 wt.%) exhibits a greater tendency to form M_6_C carbides during the solution treatment. No effects of the Fe and C content variations on the room temperature mechanical properties were observed. On the contrary, an increase in the C content resulted in a 40% lower high-temperature (760 °C) ductility of the laser powder bed fused and post-processed IN625 alloy, without affecting its strength characteristics.

## 1. Introduction

IN625 alloy is a structural alloy used in aerospace engine components and gas turbine hot-end components, such as combustion chambers, for example [1,2]. Outstanding high-temperature performances are required to overcome static, fatigue and creep loading conditions in such applications. Therefore, the aviation industry is continuously striving to improve the high- temperature mechanical properties of Inconel 625 alloy produced by laser powder bed fusion (LPBF), one of the most potent additive manufacturing (AM) processes in use. Generally, a high mechanical resistance of this alloy is the result of a solution strengthening of Nb and Fe alloying elements in the Ni-Cr matrix. Additional alloy strengthening can be obtained via the precipitation of intermetallic γ″ and δ phases [3,4]. Furthermore, the presence of Ni and Cr alloying elements improves the mechanical resistance in oxidizing environments, whereas that of Ni and Mo improves the mechanical resistance in a nonoxidizing atmosphere. Fe can also provide additional strengthening, but it renders the alloy prone to alkali and halogen attacks [5], thus reducing its corrosion resistance [6,7]. Note, however, that the formation of the δ phase must be strictly controlled because should its content increase beyond a certain level, then the ductility of IN625 decreases significantly. This phase control is complicated by the fact that the formation of δ phase is strongly processing-dependent. For example, in wrought IN625 alloys, δ phase starts to form only after prolonged aging in the 550 °C to 900 °C temperature range (10–20 h) [3,4], whereas in LPBF IN625 alloys, this phase can already be detected after only 1 h of annealing at 870 °C [8,9,10,11]. This accelerating δ phase formation is due to the fact that LPBF IN625 alloys in their as-built state have high residual stresses, fine dendritic microstructures and significant segregations of the Nb and Mo elements in the interdendritic regions [8,9,10,12].

In addition, segregation of alloying elements during the solidification of LPBF IN625 alloys can promote the formation of carbides (MC and M_6_C) and Laves phases during their post-processing heat treatments, thus decreasing the alloys’ ductility and causing instability during service at high temperatures (dynamic strain aging) [13,14]. Thus, despite the high mechanical strength of LPBF IN625 alloys at elevated temperatures (T ≥ 600 °C), their high-temperature ductility appears to be significantly lower than that of their wrought counterparts [15]. The limited elevated-temperature ductility of LPBF IN625 alloy compared to its wrought equivalent complicates the certification of LPBF IN625 alloy components and their use in the aerospace and aviation sectors [16]. To partially remedy this problem by dissolving precipitates and reducing segregations, high-temperature heat treatments can be applied to LPBF IN625 alloys [13,15,17]. This approach must, however, be applied with precaution, since the higher the annealing temperature and the longer the holding time, the coarser the microstructure and the lower the mechanical strength of LPBF IN625 alloys [15].

It can also be hypothesized that to reduce the fractions of the δ phase and carbides and the risk of strain aging, the composition of LPBF IN625 alloys must be controlled more tightly than is normally required [18]. This hypothesis is based on the following information. As shown for cast IN625, slight variations in C, Si, Nb, Mo, Fe and Ti contents within the ranges established by the IN625 alloy standards can result in different levels of risk of elemental segregation during solidification [19]. It was shown, for example, in [20] that the higher the Nb content, the higher the concentration of this element in the interdendritic regions, and of Laves phase and carbides in the solidified IN625 alloys. However, even if the Nb/Mo contents are kept near the lower bounds of their standard ranges, other alloying elements, such as Fe and Ti, could reduce the solubility of Nb and Mo in the matrix and promote their rejection into the interdendritic regions, thus intensifying the segregation phenomenon [21]. This negative effect, stemming from the presence of Fe, on the segregation of Nb and Mo, and the formation of undesirable precipitates (Laves phase, carbides) was shown in [22,23]. The impact of Fe on the solidification temperature range as well as on segregation and phase formation in weld IN625 in clad overlays fabricated on steel structures, was also studied in [11,15]. For its part, Ti promotes the formation of TiN particles, which could serve as nucleating agents for the formation of Nb and Ti carbides during solidification [24].

The effect of C and Si on phase formation during solidification of IN625 was shown in [19]. In that work, cast IN625 alloy with low C (0.009 wt.%) and Si (0.03 wt.%) contents manifested very small fractions of carbides and Laves phase as compared to the same alloy with high C (0.04 wt.%) and Si (0.46 wt.%) contents. Increasing the C content from 0.009 to 0.04 wt.%, while maintaining the Si content at 0.03 wt.%, eliminated the formation of Laves phase, but promoted that of carbides. On the other hand, increasing the Si content from 0.03 to 0.4 wt.%, while maintaining low C content (<0.01 wt.%), increased the Laves phase content and promoted the formation of large M_6_C carbides and small MC particles attached to the Laves phase.

Unlike with conventional IN625 alloys, the effect of powder composition on phase formation during additive manufacturing is not well-studied. Nevertheless, information on the subject is available. For example, it was stated in [25] that high Si and high C + N contents both favor the precipitation of the M_6_X (X = C or N) phase over the δ phase in LPBF IN625 alloy. The influence of Fe and Si contents on the formation of precipitates in IN625 alloy processed by directed energy deposition (DED) was shown in [26]. It was found that when the Si content fell below 0.05 wt.%, the Laves phase did not form in the alloy, even with the highest Fe content of 4 wt.%. On the other hand, when the Si content reached 0.05 wt.%, the volume fraction of the Laves phase increased with an increase in the Fe content from 1 to 4 wt.% [26,27]. The influence of Ti on microstructure development during laser solid forming of IN625 alloy was also shown in [28], where in contrast to wrought alloy, an increase in the Ti content up to 5 wt.% promoted the formation and growth of Laves phase inclusions at the expense of nitrides and carbides [24,28].

Two groups of works considering the segregation and precipitation phenomena in LPBF IN625 alloys also stand out: (1) works in which the segregation of Nb and Mo elements was observed, but no precipitates related to this phenomenon were found [29,30] and (2) works in which Nb- and Mo-rich precipitates were found, but their formation was not linked to a possible segregation [8,9,10]. Finally, γ″ phase and various carbides (MC, M_6_C and M_2_C) were found in the LPBF, DED and laser-cladded IN625 alloys [31,32], but the effect of powder composition on precipitation in them could not be traced due to the absence of information on the exact chemical compositions of the alloys studied. In [33], the authors used Thermo-Calc software to point out that chemical composition (variations) could result in different phase transformations and phase compositions occurring in this material. For example, it was shown that an increase in the Nb content increases the fraction of δ phase [34]. It was also shown that higher contents of Fe can result in higher fractions of Laves phase [27]. Thus, the initial powder composition of IN625 can be an important driver for a second phase formation during LPBD processing and post-heat treatments.

Different suppliers can provide IN625 powders with different chemical compositions while respecting standard specifications. It is known that IN625 alloy standards allow large variations of Cr (20–22), Mo (8–10), Nb (3.15–4.15), Fe (0–5) and C (up to 0.1) contents (all in wt.%). The purpose of this work is to obtain more information on the influence of IN625 powder composition on the microstructure and mechanical properties of LPBF components produced from this alloy. In the framework of this work, two powder compositions were studied: (1) powder with high Fe (~4 wt.%) and low C (0.02 wt.%) contents and (2) powder with low Fe (~1 wt.%) and high C (0.04 wt.%) contents, the concentrations of remaining elements in both powders being very close. It was hypothesized that increasing the Fe content will increase the degree of Nb segregation and formation of intermetallic phases, such as Laves, γ″ and δ [1]. On the other hand, increasing the C content will increase the risk of carbides formation [1]. To establish a common basis for comparison, all the specimens of this study were fabricated and heat-treated under identical conditions, their microstructures studied using the same techniques and their room and high-temperature mechanical properties measured using the same testing setups.

## 2. Materials and Methods

Two gas-atomized IN625 powders were used in this work: Powder 1 (Carpenter Powder Products, Bridgeville, PA, USA) and Powder 2 (GE Additive AP&C, Boisbriand, QC, Canada) with 15–45 µm (Powder 1) and 15–53 µm (Powder 2) particle size ranges, and D_10_ = 20 μm, D_50_ = 30 μm and D_90_ = 45 μm (Powder 1) and D_10_ = 22 μm, D_50_ = 36 μm and D_90_ = 51 μm (Powder 2) particle size distributions. For both powders, chemical compositions, particle size ranges and distributions were provided by the manufacturers in batch test certificates. The chemical compositions correspond to the UNS N06625 and ASTM F3056-14 standards (Table 1).

**Figure 1:**
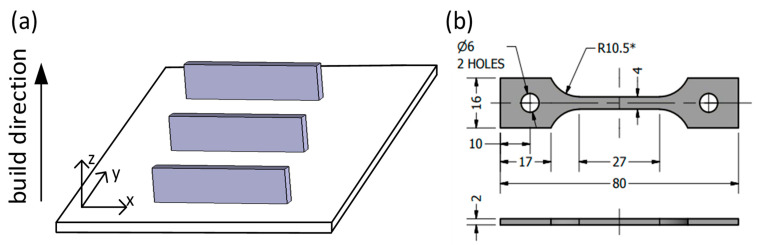
(**a**) Specimen orientations during processing; (**b**) tensile specimens machined from prismatic rectangular blanks (dimensions in mm; *—applicable for all gauge section radii).

An EOSINT M280 (EOS GmbH, Munich Germany) laser powder bed fusion system equipped with a 400 W ytterbium fiber laser was used in this work. To print IN625 specimens, EOS- recommended parameters were used: laser power ~300 W, scanning speed ~1000 mm/s, hatching space ~0.1 mm and layer thickness ~40 µm. Two print jobs were executed to fabricate horizontally oriented 85 × 18 × 4 mm^3^ blanks from two powder feedstocks (Figure 1a). This was done in an argon protective atmosphere using identical support structures and a laser scanning strategy consisting of 67° path rotations between the successive layers. Following the printing, chemical compositions were measured, and are also provided in Table 1. Note that the specimen identifications, such as Alloy 1 and Alloy 2, correspond to the powders used for their manufacture, Powder 1 and Powder 2, respectively. The alloys’ compositions were measured using 3/4″ × 3/4″ specimens and Inductively Coupled Atomic Emission Spectrometry (ICAP-AES, ASTM E1479) and Combustion (ASTM E1941) techniques with a measurement uncertainly of ≈ 1% at the 95% (k = 2) confident interval) [35].

Directly after LPBF, the blanks were cut from their platforms (stainless steel platform and 5 mm-thick IN625 support structures were used) and machined (EDM) to obtain the dumbbell-shaped tensile specimens shown in Figure 1b. Some Alloy 1 and 2 specimens were reserved to study their as-built microstructures and mechanical properties. The remaining specimens were subjected to an EOS-recommended stress relief annealing (SR) at 870 °C for 1 h with forced air cooling. The SR was performed using a Nabertherm H41/N furnace under argon continuous flow (~15 L/min). Finally, some specimens were additionally subjected to a typical high-temperature solution treatment (ST) [36] at 1120 °C for 1 h (Pyradia F200, Saint-Hubert, QC, Canada), followed by air cooling. According to [14], following this treatment, a heterogeneous columnar structure of a typical LPBF IN625 alloy (0.78 wt.% of Fe and 0.013 wt.% of C) will transform into an equiaxed structure, and this transformation is accompanied by δ phase dissolution.

The overall porosity level in printed specimens was determined using a Nikon XT-H225 computed tomography system with a 225 kV reflection X-Ray source. The specimens were scanned with a voxel size of 7 μm, a beam energy of 200 kV and a current of 50 mA using a 0.5 mm-thick copper sheet filter. The images were reconstructed using the CT PRO 3D software (Nikon Metrology Inc., Brighton, MI, USA), and the post-treatment was performed using the ORS Dragonfly image treatment software. Given such a low porosity level (less than 0.1%), its influence on the material properties were deemed insignificant and will not be discussed further. This assertion is consistent with our previous work [37] where a detailed analysis of the processing-induced porosity in IN625 alloy specimens printed using identical equipment and processing conditions showed the same level of porosity and only a few randomly distributed pores with a maximum pore size of about 60 μm with no tangible effect on the alloy’s fatigue resistance, let alone static mechanical properties.

The as-built and heat-treated (SR, ST) microstructures of the LPBF IN625 alloy specimens were studied using the X-ray diffraction (XRD, X’Pert Pro, PANalytical, Cambridge, UK) and scanning electron microscopy (SEM, Hitachi SU8230, Hitachi, Tokyo, Japan) techniques on the faces parallel (XY plan) and perpendicular (XZ plan) to the build direction. XRD analysis was performed with a Cu-Kα radiation at 40 kV and 40 mA, under a continuous scan mode over a 25–100° 2theta range. To provide a strain-free surface for the SEM (EBSD and EDX) analyses, all the specimens were polished manually (1 µm grit size), using a vibrometer and colloidal silica (0.05 μm grit size), and finally ion-milled (5 kV for 30 min). For the EBSD analysis, a high-definition e^-^FlashHR EBSD detector was used. Samples were tilted at 70° and scanned at 25 kV. For a detailed analysis of the grain structure, the EBSD scans were conducted with a step of 1 μm using four 0.4 × 0.3 mm^3^ maps for the ZX plane and two equally sized maps for the XY plane, whereas to explore the crystallographic texture, 1.5 × 2 mm^3^ maps were used. The indexing rate was ~99.8%. To evaluate the grain size, the grain boundaries and the texture, EBSD images were post-treated using the ESPRIT software (version.2.2, Bruker, Berlin, Germany) package and the HKL Channel 5 software (v.5.0, Oxford Instruments, Abingdon, UK). SEM observations of precipitates were performed using the secondary (SE) and backscattered (BSE) electron imaging modes and EDX analysis was carried out at 10 kV, using a high-sensitivity FlatQuad detector (QUANTAX FlatQUAD, Bruker, Billerica, MA, USA).

Tensile testing was conducted at 20 and 760 °C at a strain rate of 10^−3^ s^−1^ using an MTS 810 testing system equipped with an infrared radiant heating furnace. A temperature of 760 °C (1400 °F) was chosen to evaluate ductility loss commonly observed in LPBF IN625 alloys at elevated temperatures [13,14]. Elevated-temperature testing was realized under an Ar atmosphere with a flow rate of 0.1–0.3 L/min. Before testing, specimens were heated at a 1 °C/s rate and maintained at the test temperature for 10 min. Temperature control was ensured using three K-type thermocouples put in contact with the specimens’ surfaces and evenly distributed along their gauge lengths. The strains were determined using LVDT. Three tests were conducted for each processing and post-processing condition. Subsequently, the yield strength (YS), the ultimate tensile strength (UTS), and the elongation to failure (ε) were determined.

Finally, the cross-sections of specimens subjected to 760 °C testing were analyzed using EBSD with ×70 and ×300 magnifications to measure the grain size close to the fracture surfaces and to trace the crack propagation, whereas Kernel average misorientation ×300 maps were used to assess the level of internal strains.

## 3. Results

### 3.1. XRD Analysis

**Figure 2:**
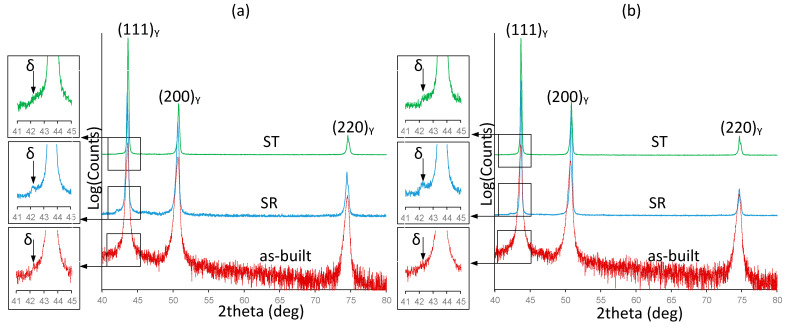
X-ray diffraction patterns of the as-built and heat-treated IN625 alloys: (**a**) Alloy 1; (**b**) Alloy 2.

Phase compositions of Alloys 1 and 2 were studied in the as-built and heat-treated states before tensile testing (Figure 2). The XRD patterns of the as-built alloys show no identifiable peaks, other than those of an FCC phase (γ matrix), confirming that the microstructure of both alloys is mainly single-phase. The fraction of other phases could be less than 1%. However, in both alloys, an additional peak belonging to the orthorhombic Ni_3_Nb δ phase appears after the stress relief annealing. After the solution treatment, the intensity of this peak was significantly reduced.

### 3.2. Precipitation and Segregation Features of the As-Built Alloy

**Figure 3:**
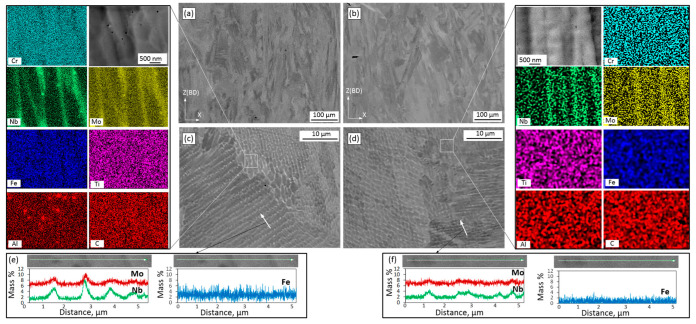
Microstructure and microsegregation patterns (in ZX plane) of the as-built alloys: (**a**,**b**) low resolution micrographs; (**c**,**d**) high resolution micrograph with elemental maps; (**e**,**f**) EDX line scans of Alloy 1 and Alloy 2.

Figure 3 shows the SEM micrographs of the vertical (ZX) planes of the as-built alloy specimens. Directional heat dissipation to the baseplate explains the formation of columnar grains along the build direction of the Alloy 1 and 2 specimens (Figure 3a,b). Figure 3c and d present high-magnification SEM micrographs of the same specimens. The solidified microstructures consist of very fine dendrites, where dark areas represent γ-matrix and the light areas represent segregation in the interdendritic regions. The average primary dendrite arm spacings are ~1.5 µm for Alloy 1 and ~1 µm for Alloy 2.

Elemental maps (Figure 3c,d) of the as-built alloys allow the determination of chemical compositions of the interdendritic and dendritic regions and the presence of some precipitated particles. In the interdendritic regions, there is obvious segregation of the Nb and Mo elements. A small number of small globular particles can also be observed in both alloys. The particle size is 60 ± 25 nm in Alloy 1 and < 60 nm in Alloy 2. According to the EDX analysis of Alloy 1, some particles are enriched in Al, some in Nb, and some in Nb/Mo, whereas in Alloy 2, their composition could not be identified because of a small size of the particles.

Figure 3e,f shows the EDX line scans and the Nb and Mo concentrations (in wt.%) in the dendritic core and interdendritic regions of both alloys. The partition coefficients k’ for both cases are calculated as the ratio of concentrations in the dendritic core and the interdendritic regions, and the smaller the partition coefficient, the greater the degree of segregation. The partition coefficients for Nb and Mo are 0.30 ± 0.08 and 0.80 ± 0.05 for Alloy 1 and 0.60 ± 0.06 and 0.92 ± 0.02 for Alloy 2, respectively. Thus, under the same processing conditions, Alloy 1 manifests a more intensive segregation of the Nb and Mo elements during solidification than Alloy 2. These differences in the element segregation intensity could contribute to the differences seen in the precipitation kinetics, size, morphology and number of precipitates. Contrary to the Nb and Mo elements, Fe exhibits higher concentrations in the dendritic core as compared to the interdendritic regions. The partition coefficients k’ is in this case 1.2 and 1.3 for Alloys 1 and 2, respectively.

### 3.3. Effect of SR and ST on the Precipitation Distribution and Morphology

**Figure 4:**
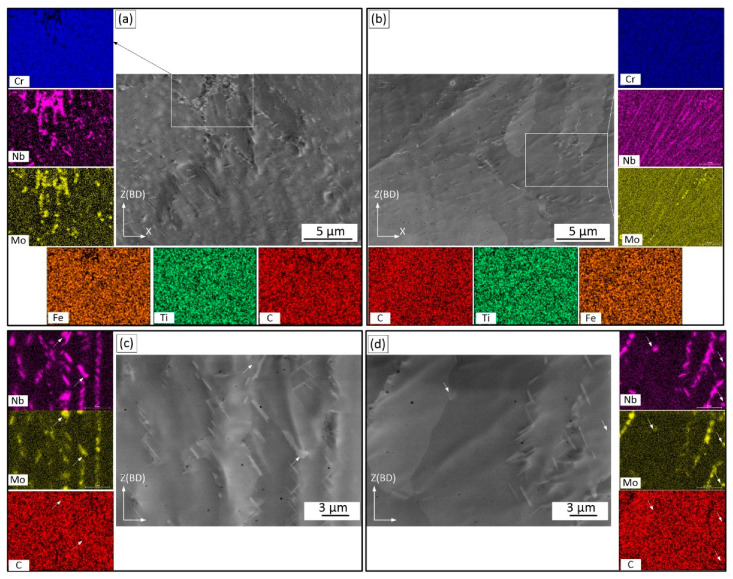
Precipitation after SR in (**a**,**c**) Alloy 1 and (**b**,**d**) Alloy 2 with their respective elemental maps.

After the SR treatment, precipitation distributions throughout the matrices of both alloys are shown in the SEM micrographs of Figure 4. It can be seen that the precipitates are mainly aligned along the interdendritic regions.

Figure 4c,d shows high magnification images of particles in Alloys 1 and 2. The microstructures of both alloys contain predominantly needle- and plate-like particles. In Alloy 1, the thickness of the plate-like particles is approximately several tens of nanometers, with the length varying from 1 to 3 µm. In Alloy 2, the particles are smaller in size. The EDX maps illustrate that the precipitates are enriched in Nb and Mo (Figure 4c,d). Very fine spherical precipitates, also enriched in Nb and Mo, can also be observed. The Mo concentration in these particles is higher than in the plate-like particles. Note that in LPBF IN625 alloy subjected to SR at 870 °C, particles with similar morphologies, compositions and proportions of Nb and Mo elements were identified as orthorhombic Ni_3_Nb δ phase (plate-like particles) and M_6_C carbides (globular particles) [11,12,29,33]. In summary, after SR, Alloy 1 contains larger and less uniformly distributed particles than Alloy 2 (Figure 4c,d), which can be attributed to a more heterogeneous chemical composition of the former as compared to the latter.

**Figure 5:**
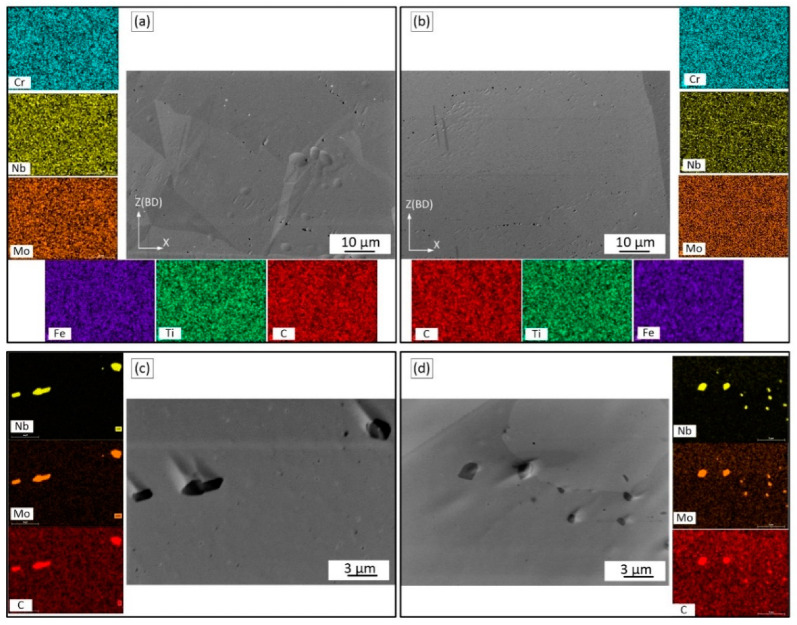
Precipitation after the ST in (**a**,**c**) Alloy 1 and (**b**,**d**) Alloy 2, with their respective Nb and Mo elemental maps.

The SEM micrographs of both alloys after the solution treatment are shown in Figure 5. The columnar dendritic solidification structures and the δ precipitates can no longer be observed, because the solution treatment homogenized the Nb and Mo elements distributions, or probably because they are too small to be observed using SEM. However, small spherical particles can still be observed in both alloys, and they manifest two types of distributions: (1) linear-chain distribution, resembling the characteristic features of as-built and stress-relieved alloys; and (2) random distribution throughout the matrix. Some of the randomly distributed particles are located along the grain boundaries and can be referred to as “secondary” precipitates. It is also observed that the chain particles are coarse, with an average size of 3 µm, whereas the other particles are much smaller (~100 nm). According to the EDX analysis, all the particles are enriched in Nb and Mo. Additionally, coarse chain particles clearly contain C, and so they can be recognized as M_6_C carbides, whereas the same conclusion cannot be drawn for small randomly distributed particles since their EDX intensity is not sufficiently high. Overall, Alloy 1 contains a smaller carbide fraction (0.2 ± 0.2%) than Alloy 2 (0.5 ± 0.3%).

### 3.4. EBSD Analysis of the As-Built and Heat-Treated Alloys

**Figure 6:**
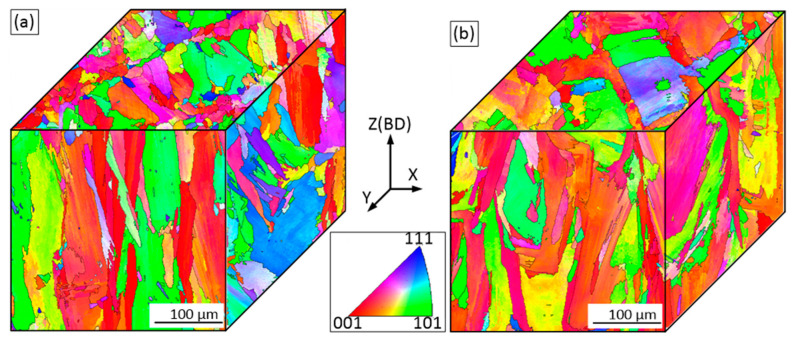
Orientation image microscopy (OIM) maps for the ZX and XY planes of the as-built (**a**) Alloy 1 and (**b**) Alloy 2.

The EBSD maps allow the assessment of grain orientations, morphologies and textures in the planes parallel and perpendicular to the build direction, BD (Figure 6, color coding is applied to reveal the crystal orientation in BD). It can be seen that the grains are indeed elongated and that the grain growth direction is approximately parallel to the BD. The grains are not uniform in size, and fine and coarse grains coexist. The columnar grain geometry can be described by two values: average length (L_Z_) and average width (L_X_). Additional information on the grain shape can be obtained from the grain aspect ratio (AR) or the anisotropy index (AI) as AR = AI = L_Z_/L_X_ (ASTM E112-13). (Note that more than 300 grains were counted for each alloy). In the ZX plane, the average grain width and length of the as-built Alloy 1 are 15 ± 15 and 43 ± 42 µm, whereas the average grain width and length of the as-built Alloy 2 are 16 ± 14 and 48 ± 46 µm, respectively. Consequently, in the ZX plane, the AI value is 2.9 for Alloy 1, and 3.0 for Alloy 2, this difference being statistically insignificant.

Variations in grain size are found in the perpendicular XY plane as well. Fine equiaxed grains are spotted around larger grains and follow the lines corresponding to the laser overlap regions. For both alloys, the distance between these lines is close to the hatching space (100 µm) used during LPBF processing. Since grains in the XY plane are near-equiaxed, their size can be calculated using a circle equivalence method (ASTM E 112). The average grain size in the XY plane is 17 ± 13 µm for Alloy 1 and 18±15 µm for Alloy 2, this difference being statistically insignificant.

**Figure 7:**
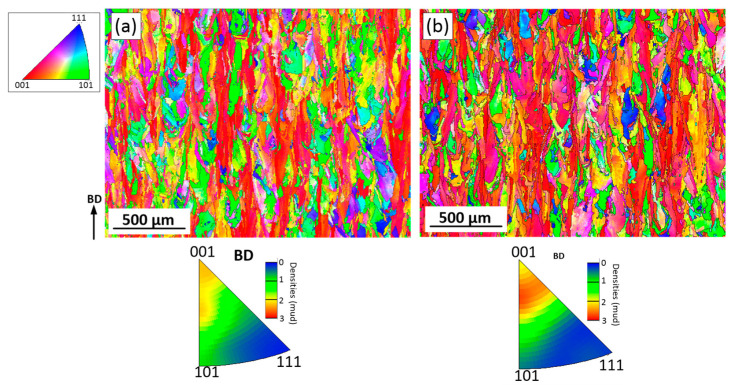
Orientation image microscopy (OIM) maps and inverse pole figures (IPFs) in the ZX plane of the as-built (**a**) Alloy 1 and (**b**) Alloy 2.

Since the microstructures of the as-built LPBF alloys are heterogeneous, inverse pole figures (IPFs) were constructed using the EBSD maps counting more than 2000 grains. Figure 7 shows the EBSD maps and the IPFs in the principal direction (BD or the ZX plane). It can be seen that LPBF processing promotes the formation in both alloys of a <100> texture, and this texture is slightly weaker in Alloy 1 than in Alloy 2.

**Figure 8:**
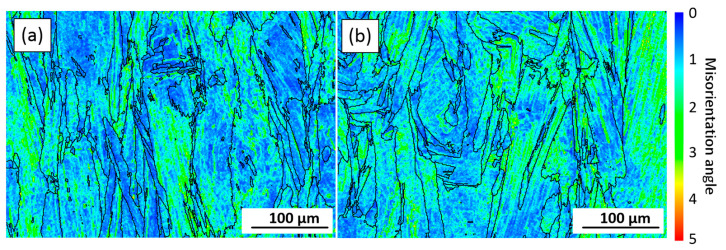
KAM maps (ZX plane) of the analyzed areas in the as-built (**a**) Alloy 1 and (**b**) Alloy 2.

Kernel average misorientation (KAM) maps obtained from the EBSD data (Figure 6) illustrate changes in local misorientations caused by geometrically necessary dislocations (Figure 8) [38]. The KAM values also reveal areas with increased defect density and reflect the level of local strains [39]. In Figure 8, blue and red colors represent the lowest and the highest strain/dislocation densities, respectively. Heterogeneous distributions of the strain/dislocation density are presented in both alloys, and higher strain/dislocation densities are observed in elongated grains. For both alloys, average KAM values were calculated and correspond to 1.12° ± 0.03° for Alloy 1 and 1.22° ± 0.04° for Alloy 2, which indicates a slightly higher strain/dislocation density in the latter. The analysis of variance (ANOVA) showed a statistically significant difference between the mean values (*p*-value < 0.05).

**Figure 9:**
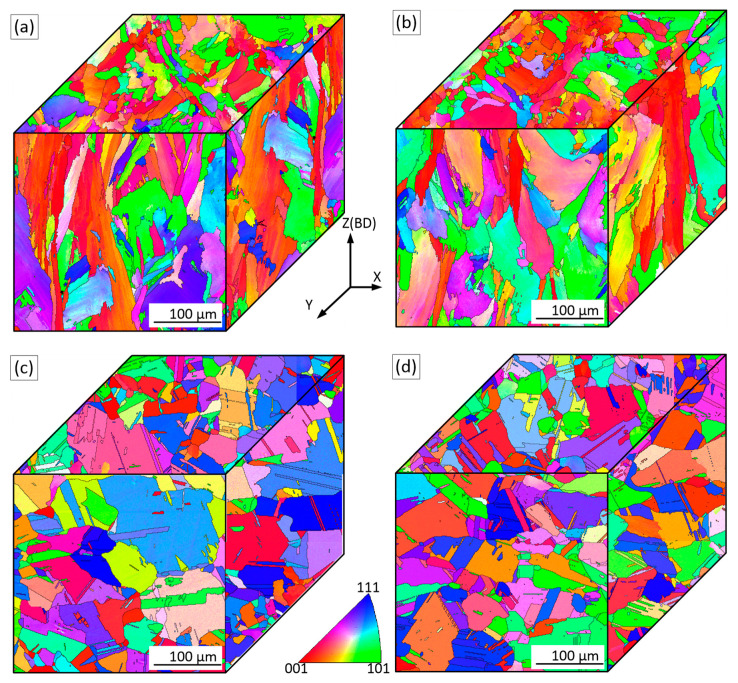
Orientation image microscopy (OIM) maps for ZX and XY planes: (**a**,**b**) after SR treatment; (**c**,**d**) after ST treatment; (**a**,**c**) Alloy 1 and (**b**,**d**) Alloy 2.

**Figure 10:**
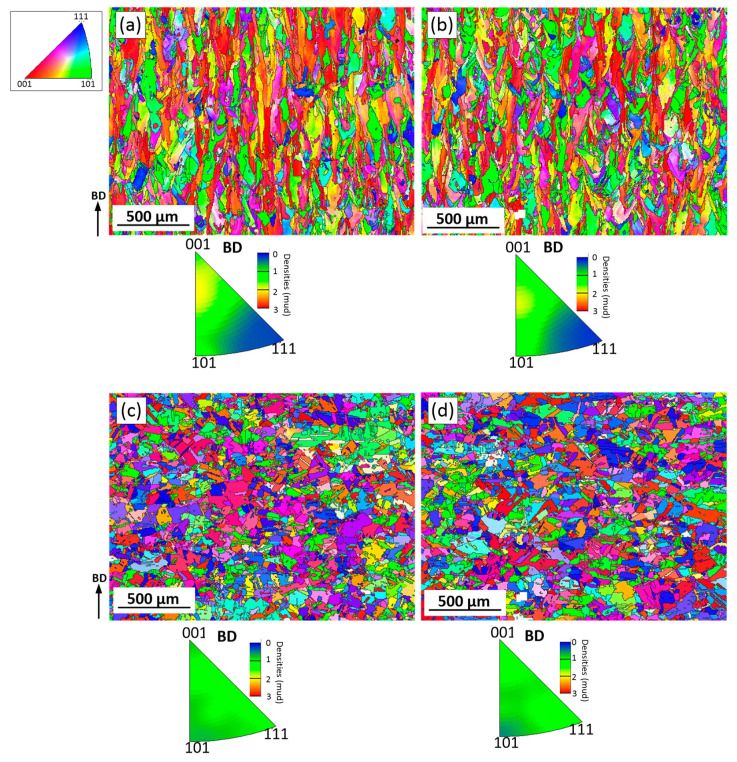
Orientation image microscopy (OIM) maps and inverse pole figures in the ZX plane (IPFs) of (**a**,**c**) Alloy 1 and (**b**,**d**) Alloy 2. (**a**,**b**) after SR; (**c**,**d**) after ST.

The EBSD images of Alloys 1 and 2 after SR and ST treatments are presented in Figure 9. It can be seen that after the SR treatment, the grains are still elongated, and their average width and length are 17.5 ± 17 and 42 ± 38 µm for Alloy 1, and 18 ± 16 and 43 ± 36 µm, for Alloy 2. Thus, the AI values of both alloys are similar and equal to ~2.4. An average grain size in the XY plane, calculated using a circle equivalence method, is 17 ± 14 µm for Alloy 1 and 18 ± 13 µm for Alloy 2. The < 100 > texture is still the preferred crystallographic orientation in the build direction, but it becomes obviously weaker after the heat treatments (Figure 10a,b).

It can be seen that the ST leads to the recrystallization of both alloys: grains become semi-equiaxed and no preferred crystal orientation can now be observed (Figure 10c,d). The appearance of annealing twins also indicates that the recrystallization process takes place during the ST of both alloys. In the ZX plane, the average grain size, including twin boundaries, is 24 ± 24 µm for Alloy 1 and 31 ± 38 µm for Alloy 2. The grain size excluding the twin boundaries is 54 ± 90 µm for Alloy 1 and 68 ± 100 µm for Alloy 2. In the XY plane, the average grain size of Alloy 1 is 28 ± 29 and 57 ± 43 µm, with and without twin boundaries, respectively. The average grain size of Alloy 2 is 30 ± 20 and 72 ± 71 µm with and without twin boundaries, respectively.

**Figure 11:**
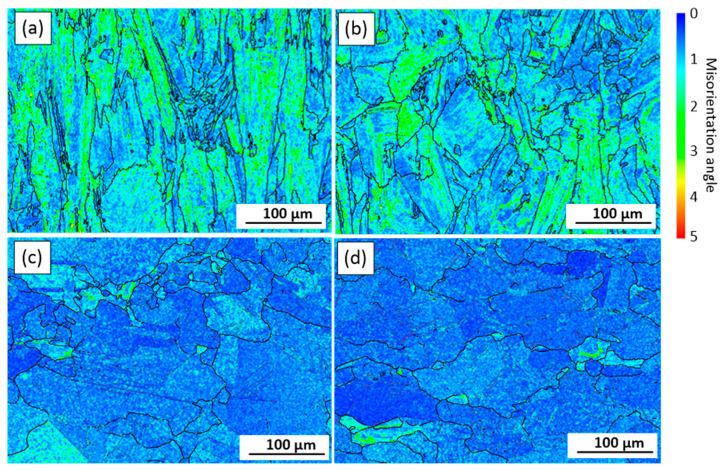
KAM maps (ZX plane) after (**a**,**b**) SR and (**c**,**d**) ST treatments of (**a**,**c**) Alloy 1 and (**b**,**d**) Alloy 2.

A slight decrease in the strain/dislocation density can be observed after the SR treatment as compared to the as-built state, for both alloys (Figure 11a,b). After SR, the KAM values become 1.18° for both alloys, whereas after ST, these values decrease to 0.63°. However, the strain is not fully removed after the ST treatment (Figure 11c,d), and although the dislocation density decreases, some fine grains still contain high dislocation densities.

After ST, the HAGB (high-angle grain boundary) densities (including twin boundaries) increase from ~110 to ~140 mm^−1^ for both alloys as compared to their as-built state. Generally, the HAGB can effectively arrest microcrack propagation, thereby enhancing material toughness [40]. Additionally, twinning boundaries could improve the material strength without decreasing the elongation at room and elevated temperatures [41,42,43]. Thus, based on the microstructure features, a higher toughness can be expected from the ST alloys as compared to their as-built and stress-relieved counterparts.

### 3.5. Mechanical Properties

**Figure 12:**
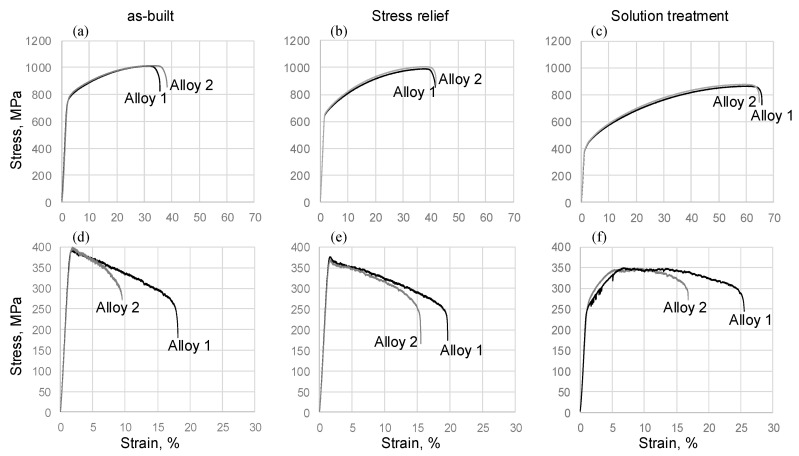
Tensile behavior of Alloys 1 and 2 at (**a**–**c**) room temperature and (**d**–**f**) 760 °C; (**a**,**d**) the as-built state, (**b**,**e**) after the SR and (**c**,**f**) after the ST treatments.

Figure 12 shows typical tensile stress-strain curves of the two alloys at 20 and 760 °C. It can be seen that at room temperature, the tensile behaviors of both alloys in the as-built state and after SR and ST treatments are similar, and do not therefore manifest any compositional dependency. As expected, the as-built alloys exhibit the highest yield stress (YS) and ultimate tensile strength (UTS) values and the lowest elongations to failure (Table 2). After the SR treatment, the YS and UTS values decrease and reach their lowest values after the ST treatment, whereas the elongations manifest an opposite trend, with the highest values reached after the ST treatment.

Contrary to the preceding, at 760 °C, the stress-strain curves of Alloys 1 and 2 differ significantly. Although the strength characteristics of both alloys are similar and exhibit the same trends (highest YS and UTS in the as-built state and lowest equivalents in the SR and ST states), significant differences are observed in their high-temperature elongations. At 760 °C, Alloy 1 in the as-built state and after SR and ST treatments shows significantly higher elongations to failure than Alloy 2. Moreover, at 760 °C, both alloys in the as-built state and after the SR exhibit yield peaks followed by a work softening down to failure. After the ST, however, both alloys show a significant work hardening, but this behavior is accompanied by serrations for only one of them, namely, Alloy 1.

### 3.6. EBSD Observations after High-Temperature Tensile Testing

**Figure 13:**
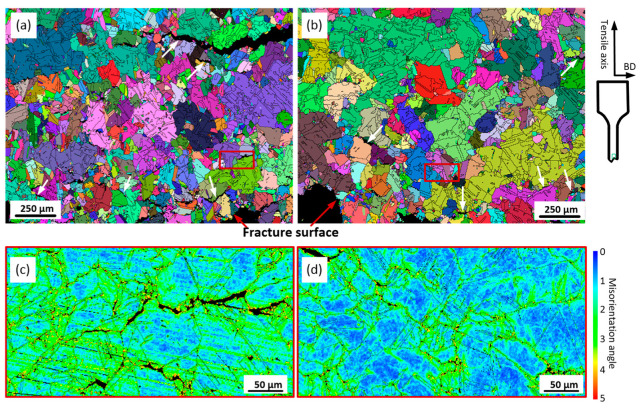
Grains under the fracture surfaces (longitudinal cross section) of (**a**) ST Alloy 1 and (**b**) ST Alloy 2 after tensile testing at 760 °C (white arrows indicate microcracks); KAM maps for selected area of (**c**) ST Alloy 1 and (**d**) ST Alloy 2.

The longitudinal cross-sections (i.e., along the tensile axis) of ST specimens subjected to tensile testing at 760 °C were analyzed and the results are shown in Figure 13. Note that the grains are in random color. From these observations, in addition to the main crack, some secondary cracks underneath and parallel to the fracture surface can be seen in both alloys. The EBSD maps show the propagation of the cracks along the grain boundaries. It is clear that the grain boundaries of both alloys are weaker as compared to the grain interiors and twins HAGBs.

The grains located closer to the fracture surface are smaller than those located farther from it. For example, an average grain size close to the fracture surface of both alloys is 17 ± 2 µm (AR = 1.3), whereas an average grain size from the adjacent area is 18 ± 2 µm (AR = 1.4) for Alloy 1 and 24 ± 4 µm for Alloy 2. Thus, after tensile testing, the average grain size of Alloy 1 decreases from 54 to 32 µm, and for Alloy 2, from 68 to 33 µm, as compared to their sizes before mechanical testing (note that these values were calculated for a total area of 1.5 × 2 mm^2^). These observations indicate that during tensile testing, dynamic recrystallization occurs in the areas close to the fracture surfaces of both alloys. Moreover, during tensile testing of both alloys, high strain levels are accumulated along the HAGBs (Figure 13c, d). Intergranular cracking of Alloy 2 occurs at lower strain levels that those of Alloy 1, probably due to a higher number of carbide particles along the grain boundaries. Thus, the grain boundaries of Alloy 1 apparently provide higher resistance to cracking than those of Alloy 2.

### 3.7. Fractography

**Figure 14:**
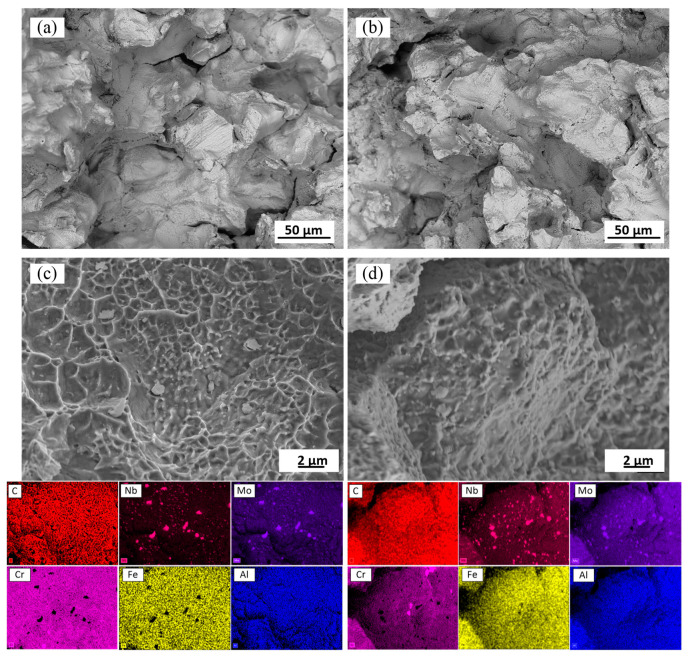
Fracture surface of the solution-treated Alloy 1 (**a**,**c**) and Alloy 2 (**b**,**d**) after tensile testing at 760 °C; EDX elemental maps.

Figure 14 shows the fracture surfaces of both solution-treated alloys after tensile testing at 760 °C. It is evident that during tensile testing at elevated temperatures, cracks propagate along the grain boundaries of both alloys. Many secondary cracks can also be observed. The secondary crack formation is due mainly to structural heterogeneity. The fracture surfaces consist of the grain boundary voids formed during plastic deformation. The voids coalesce to form the grain boundary cracks. Some voids appear in the vicinity of grain boundary precipitates (Figure 14). Many particles are rich in Nb and Mo, whereas few are rich in Cr. The carbon was probably not revealed because of its low content in M_6_C and M_23_C carbides and the complex topography of fracture surfaces. However, it can be seen once again that on the fracture surface, the volume fractions of the Nb and Mo-containing particles in Alloy 1 are relatively lower than those in Alloy 2.

## 4. Discussion

IN625 specimens were fabricated using powder feedstocks with ~1.0 and ~4.0 wt.% of Fe (near the extremes of the allowable composition range) and subjected to stress relief annealing at 870 °C and then to a solution treatment at 1120 °C. The low Fe content powder contained twice as much carbon (~0.04 wt.%) as the high Fe content powder (~0.02 wt.%). Whereas the Fe influenced the segregation of the Mo and Nb elements in the interdendritic regions of γ-phase during printing and the formation of δ phase during stress relief annealing, carbon, in turn, influenced the formation of carbides during the solution treatment. Thus, the effect of both elements on the microstructure and mechanical properties of LPBF IN625 alloy was studied in this work.

*As-built structure*: According to the results, columnar grains with a dendritic structure were formed in both alloys because of the high thermal gradients and solidification rates accompanying the LPBF process. For both alloys, the grain size, morphology and orientation were found to be similar. The primary dendritic spacing of about 1–1.5 µm was found consistent with [29,44,45], and the grain size of 15–16 µm in width, and 43–48 µm in length, were found similar to [46]. In both alloys, solute elements were not completely trapped in growing crystals, thus resulting in the segregation of the Nb and Mo elements. Note that the partition coefficients k’ for Nb and Mo of the two alloys were found to be significantly different. Alloy 1 manifested higher segregation than Alloy 2 because of its higher Fe content and therefore lower solubility of Nb and Mo elements in the γ matrix [26,47,48]. This strong segregation is deemed responsible for the formation of small precipitates in the as-built Alloy 1. Similar small particles (10–50 nm) were also found in the as-built LBPF IN625 alloys, and they were identified as NbC carbides in [44].

*Heat-treated structure*: Segregation of Nb and Mo elements in the interdendritic regions was found to be responsible for the formation of δ precipitates in these regions during stress relief annealing at 870 °C, similarly to what was observed in weld IN625 alloys [3]. A higher Nb content in the interdendritic regions of Alloy 1 promoted the formation of coarser and less uniformly distributed δ particles as compared to Alloy 2. It is believed that a heterogeneous distribution of Nb/Mo in the as-built Alloy 1 led to the heterogeneous precipitation of the δ phase during the SR annealing. In addition to the δ phase, small spherical Nb/Mo-containing particles were also seen in the interdendritic regions of both alloys. The chemical composition of these particles differs from that of the δ phase, and they were identified as M_6_C carbides, similar to Nb/Mo-containing particles in the stress-relieved LPBF IN625 [13].

A high-temperature solution treatment at 1120 °C led to the dissolution of the δ phase and the chemical homogenization of both alloys. However, non-uniformly distributed M_6_C carbides initially formed in the interdendritic regions during the SR annealing grew during the solution treatment to reach ~3 µm in size. Secondary fine carbides were also observed along the grain boundaries after this treatment and Alloy 2 contained twice as many carbides as Alloy 1 (0.5% versus 0.2% in volume, ASTM E562). The ST treatment also resulted in the dislocation density reduction and the formation of annealing twins and near-equiaxed grains during the recrystallization process. In Alloy 1, the grain size became 57 ± 43 and 54 ± 90 µm in the XY and ZX planes, whereas in Alloy 2, it became 72 ± 71 and 68 ± 100 µm, respectively.

**Figure 15:**
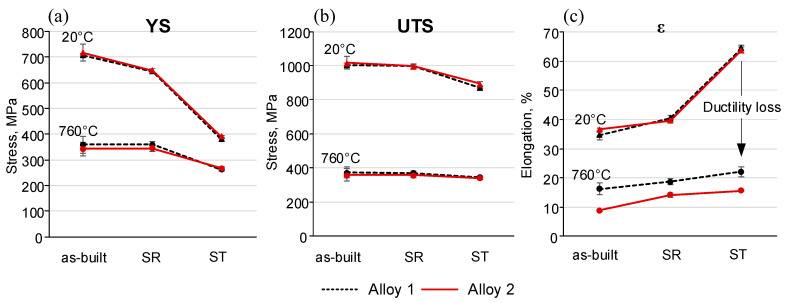
Mechanical properties (at 20 °C and 760°) of Alloys 1 and 2 in the as-built state and after the SR and ST treatments: (**a**) yields stress (YS), (**b**) ultimate tensile strength (UTS) and (**c**) elongation to failure (ε).

*Mechanical properties*: Despite the differences in the alloy compositions, the mechanical properties at room temperature of both alloys were very close (Figure 15). At room temperature, Alloy 2 (with a lower Fe content) showed a slightly higher mechanical strength as compared to Alloy 1. This observation is not in agreement with [26], where the directed energy deposited (DED) IN625 alloy with a low Fe content (1 wt.%) demonstrated a significantly finer microstructure and higher YS and UTS values as compared to the same alloy with a high Fe content (4 wt.%). However, the mechanical strength (YS = 710–720 MPa and UTS = 1005–1020 MPa) of the LPBF IN625 alloys of this study is higher than that of the DED IN625 alloy (YS = 450–520 MPa and 750–860 MPa) [26], and close to what was given for the LPBF IN625 alloy in [49].

At room temperature, the as-built alloys showed the highest mechanical strength. The SR treatment slightly reduced the mechanical strength characteristics (YS, UTS) of both alloys, and the solution treatment led to their lowest mechanical strength due to δ phase dissolution and microstructure coarsening (Figure 15a,b). At 760 °C, as at room temperature, the strength characteristics of both alloys were similar. The as-built and stress-relieved alloys exhibited work softening after a stress peak value of ~0.3%, with close YS and UTS values for both alloys. After the solution treatment, the YS values of both alloys decreased significantly, whereas the UTS values remained high due to work hardening. At 760 °C, Alloy 1 showed serrations, thus indicating the possible presence of dynamic strain aging, whereas no serrations were observed in Alloy 2. The resistance of Alloy 2 to dynamic strain aging is attributed to a higher fraction of fine carbides found in this alloy. This assertion is supported by the observations in [50], where the dynamic strain aging effect was significantly reduced by closely spaced secondary phases, preventing the critical carbon build-up needed for dislocation locking and unlocking (serrations).

At room temperature, the elongations to failure of both alloys were close, and the values increased after the heat treatments (Figure 15c). On the contrary, at 760 °C, the elongations to failure decreased significantly, especially after ST. Thus, the two alloys manifested the so-called elevated temperature ductility loss ($\Delta \varepsilon =\varepsilon_{20{}^{\circ}C}-\varepsilon_{760{}^{\circ}C}$) observed in many Ni-based alloys [51,52,53,54,55,56,57,58,59,60,61]. This ductility loss reached its maximum after the solution treatment, and despite the dissolution of the δ phase, chemical homogenization and microstructure coarsening resulted from the treatment. This high-temperature ductility loss was accompanied by intergranular crack propagation, and numerous particles enriched in Nb and Mo elements were found on the fracture surfaces of both alloys. These observations are consistent with those in [13], where cracks were found to propagate along grain boundaries containing M_6_C carbides. The solution-treated Alloy 2 specimens contained a higher volume fraction of M_6_C carbides, which resulted in their lower high-temperature ductility.

To sum up, Alloys 1, with a higher Fe content (4.28 wt.%) manifested a greater propensity for segregation and the formation of heterogeneous microstructures and the δ-phase. To decrease the risk of segregation in the LPBF IN625 alloys, it can be recommended to keep the Fe content in feedstock powders as low as possible. Despite the negative segregation-related phenomena observed in Alloy 1, which stemmed from the high Fe content, the alloy demonstrated a significantly higher elevated temperature ductility in comparison with Alloy 2. The reason for this discrepancy is that a higher C content in Alloy 2 (0.035 wt.%) led to a higher concentration of M_6_C carbides and a lower resistance of its grain boundaries to cracking. A significant number of fine carbide particles in Alloy 2 disrupted the accommodation mechanisms restricting the mobility of extrinsic dislocations along the grain boundaries [62]. As shown in [63], the negative impact of C on the high-temperature properties of IN625 can be avoided by lowering the carbon content to the 0.015–0.025 wt.% C range.

Even though a tight control of the C content could reduce the amount of carbides and improve the ductility of LPBF IN625 alloys, increasing the solution treatment temperature beyond 1120 °C can equally be recommended to promote both alloy homogenization and carbide dissolution. This recommendation is supported by the findings in [49], where a high-temperature solution treatment at 1300 °C for 1.5 h followed by aging at 870 °C for 2 h led to significant changes in the mechanical behavior of LPBF IN625 alloy: no ductility loss was observed in this alloy at 700 °C as compared to its as-built state.

## 5. Conclusions

The effect of powder composition on the microstructure and mechanical properties of IN625 were investigated. The following main conclusions can be drawn:

- A higher content of Fe results in a higher level of segregation in the interdendritic regions of the as-built IN625 alloy, with the grain size not being affected by this.
- More intensive segregation in the IN625 alloy with a higher Fe content leads to an enhanced formation of the δ phase and carbides during stress relief annealing at 870 °C.
- The Fe content has no effect on the carbide formation during solution treatment at 1120 °C, whereas the higher the C content, the higher the fraction of carbides formed during this treatment.
- The room temperature mechanical properties of the LPBF IN625 alloy were found insensitive to the Fe and C content variations studied in this work; the effect of processing overcame that of chemical composition. On the contrary, a higher C content in one of the IN625 powders resulted in a greater fraction of fine M_6_C carbide particles at the grain boundaries and a lower high-temperature ductility of the LPBF IN625 alloy. High-temperature ductility of the LPBF IN625 alloy was found insensitive to the Fe content variations studied in this work.

## Figures and Tables

**Table 1:** Chemical compositions of the IN625 specimens and of standard IN625 alloy (in wt.%).

IN625	Ni	Cr	Mo	Nb	Fe	Ti	Al	C	O	Si	S
**Standard**	Bal	20–22	8–10	3.15–4.15	<5	<0.4	< 0.4	<0.1	n/a	<0.5	<0.015
**Powder 1 (15–45 µm)**	Bal	20.8	9.1	3.64	4.2	0.21	0.21	0.02	0.014	0.08	<0.015
**Alloy 1 (from Powder 1)**	Bal	21.15	9.8	3.68	4.28	0.19	0.21	0.024	-	0.07	<0.001
**Powder 2 (15–53 µm)**	Bal	21.66	8.86	3.66	0.95	0.26	0.15	0.040	0.011	0.07	<0.001
**Alloy 2 (from Powder 2)**	Bal	21.89	9.93	3.79	0.97	0.12	0.13	0.035	-	0.03	<0.001

**Table 2:** Mechanical properties of the LPBF IN625 alloys.

State	Properties	Alloy 1		Alloy 2	
		20 °C	760 °C	20 °C	760 °C
**as-built**	YS, MPa	710 ± 5	360 ± 30	720 ± 20	340 ± 30
	UTS, MPa	1005 ± 10	370 ± 30	1020 ± 30	360 ± 40
	Elongation, %	35 ± 2	16 ± 2	37 ± 1	9 ± 1
**SR**	YS, MPa	645 ± 10	360 ± 15	650 ± 10	340 ± 10
	UTS, MPa	1000 ± 15	370 ± 10	1000 ± 10	360 ± 15
	Elongation, %	40 ± 1	19 ± 1	40 ± 1	14 ± 1
**ST**	YS, MPa	380 ± 10	260 ± 5	390 ± 5	270 ± 10
	UTS, MPa	870 ± 5	345 ± 10	890 ± 15	340 ± 10
	Elongation, %	65 ± 1	22 ± 2	64 ± 1	16 ± 1

## Data Availability

Not applicable.
